# Supplementary material for: iSeg: an efficient algorithm for segmentation of genomic and epigenomic data
Source: BMC Bioinformatics. 2018 Apr 11;19:131. doi: 10.1186/s12859-018-2140-3 (PMC5896135; doi:10.1186/s12859-018-2140-3)
Supplement: Supplementary file 1 — The zipped supplementary file submitted with this manuscript contains Coriell (Snijders et al.) profiles, BACarray profiles, and simulated data files. (ZIP 1970kb) [file 12859_2018_2140_MOESM1_ESM.zip › SF3-ParSettings.docx]

In our analysis, **fdr** and **alp** are parameters we used to assess segments.

**Coriell Dataset**

- Default settings for all methods.
- They all used a 0.05 level of significance to assess each segment.

**Simulation**

- DNACopy: sig = 0.01, fdr = 0.01, alp = 0.05, undo = “none”, trim = 0.025, sdundo = 3, undo.prune = 0.05
- HMMSeg: Default settings, required alp = 5e-10 cut-off to determine significant segments.
- CGHSeg: sig = 0.00001, fdr = 0.001, alp = 0.01, nbl = 3, sel = "none", pos = "none"
- Default settings for other methods

**BAC**

- FastSeg: sig = 1e-4, fdr = 0.01, alp = 0.05, mseg = 4, cyberWeight = 10, squashing = 0. Here, ‘sig’ is our level of significance and ‘alp’ is the internal parameter for alpha in FastSeg.
- CGHSeg: alp = 0.1, nbl = 3, fdr = 0.01, sig = 0.001. Other parameters at Default.
- HMMSeg: alp = 0.0005; Other parameters at Default.
- CGHFLasso: fdr = 0.01 and sig = 0.001; Other parameters at Default.
- Other methods had Default Settings.

**Long Sequence Simulation**

- DNACopy: sig = 0.01, fdr = 0.01, alp = 0.05, undo = "none"; Other parameters at Default.
- HMMSeg: alp = 0.05
- CHSeg: alp = 0.00001, sig = 0.01, fdr = 0.001, nbl = 3, sel = "none", pos = "none"; Other parameters at Default.
- CGHFLasso: Default settings.

**Segmentation of single nuclease sensitivity profile**

- iSeg: fdr = 0.05; bc = 1.0, 2.0, 3.0.
- MACS: fdr = 0.05(default), 0.01, 0.001; local lambda estimate.
- SICER: fdr = 0.05(default); (window, gap) = (20, 40), (30, 60), (50, 100).

**Segmentation of difference profiles with both positive and negative values**

- iSeg: fdr = 0.05; bc = 1.0, 2.0, 3.0.
- MACS: fdr = 0.05(default), 0.01, 0.001; local lambda estimate.
- SICER: fdr = 0.05(default); (window, gap) = (20, 40), (30, 60), (50, 100).
- PePr: fdr = 0.05(default); mode: broad peak, narrow peak.
